# Foliar Anatomy of Three Native Species of *Tillandsia* L. from the Atacama Desert, Chile

**DOI:** 10.3390/plants11070870

**Published:** 2022-03-24

**Authors:** Eliana Belmonte, Bernardo Arriaza, Mabel Arismendi, German Sepúlveda

**Affiliations:** 1 Departamento de Biología, Facultad de Ciencias, Universidad de Tarapacá, Arica 1000007, Chile; 2 Instituto de Alta Investigación, Universidad de Tarapacá, Arica 1000007, Chile; barriazaarica@gmail.com; 3 University of California Davis Chile Life Sciences Innovation Center, Av. Santa María 2670, Tower B, of. 206, Providencia, Santiago 7520424, Chile; arismendimabel@gmail.com; 4 Departamento de Recursos Ambientales, Facultad de Ciencias Agronómicas, Universidad de Tarapacá, Arica 1000007, Chile

**Keywords:** Atacama, Chile, peltate trichomes, desert vegetation

## Abstract

In the extreme north of Chile, the genus *Tillandsia* L. (Bromeliaceae) is represented by three native species, *T. marconae* Till & Vitek and *T. landbeckii* Phil., both of terrestrial atmospheric habit, and *T. virescens* Ruiz & Pav. of saxicolous habit. There is little information on the foliar structures that allow its establishment in arid environments. Therefore, we studied the leaf anatomy of each of these terrestrial and saxicolous atmospheric species from different altitudinal levels (1000 and 3000 m) in the Arica and Parinacota regions of the Atacama Desert. All populations are monospecific. The study considered scanning electron microscopy, optical microscopy, and the fingernail polish technique. The surface distribution of stomata and trichomes of the species is described. The studied species presented hypostomatic leaves, with anomocytic stomata and peltate trichomes on the abaxial and adaxial sides. Trichomes are formed by a central disc of four equal-sized empty cells, surrounded by a peripheral series of several concentric rings, the innermost of eight, the second of sixteen and the outermost of multiple elongated cells, one cell thick, that form the flexible asymmetric wings. The number of wing cells varies according to the species. Trichomes are evenly arranged in long lanceolate leaf blades with smooth margins.

## 1. Introduction

*Tillandsia* is a genus of Bromeliaceae [1,2] with epiphytic, atmospheric terrestrial, and saxicolous plants [3,4]. In recent years, several new species have been described [5,6,7], and some anatomical adaptations have been studied that make it successful in the difficult environment of the Atacama Desert [8].

**Figure 1:**
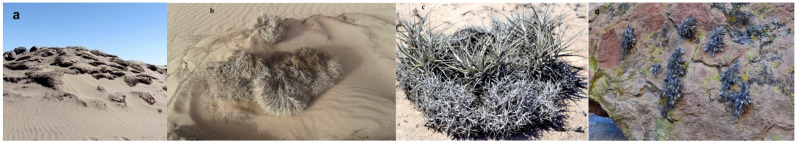
*Tillandsia* in situ. (**a**,**b**). *Tillandsia landbeckii* mounds (Pampa Camarones 1010 m). (**c**). *Tillandsia marconae* growing next to *T. landbeckii* (Pampa Dos Cruces 1000 m). (**d**). *Tillandsia virescens*, saxicolous species, hanging from a rock (Pukara de Copaquilla 3000 m).

In northern Chile, tillandsias are described as plants dependent on fog, for which they have developed a complex system of foliar trichomes with which they collect water from the fog or “camanchaca”, a typical name of northern Chile [3,9,10,11,12]. They are sustained on sandy soil, growing in vegetative bands of approximately 40–60 cm wide by 1 to 2 m long or more and can form mounds of varying heights, some of which exceed 2.5 m high by 10 or more m long, which are formed by superimposing bands separated by sterile material [13,14]. The survival strategy of these plants involves a series of events, from the implantation of a new shoot and the successive accumulation of edaphic material with the development of new shoots, generating a growth stand, which is later projected as a community of plants [15] (Figure 1). 

In Chile, the spatial site of these populations [14], the ecosystems [16], and the main macroscopic plant and animal species associated with them [17,18] have been described. 

The Atacama Desert is characterized by low species diversity in areas of hyper aridity [19,20,21,22]; regardless, the best-represented terrestrial atmospheric species between 900 and 1300 m are *Tillandsia landbeckii* Phil. and *T. marconae* Till & Vitek [23]. *T. virescens* Ruiz & Pav., an atmospheric saxicolous species that develops profuse short adventitious roots, inhabits the cliffs around the locality of Copaquilla, at 3000 m. These three species are native [24]. The location of *T. marconae* is unique in Chile, being found only in Pampa Dos Cruces, 30 km inland from Arica, where it shares space with *T. landbeckii*.

*Tillandsia marconae* and the population of *T. landbeckii* used in this study are found on plateaus encountered at similar altitudes (Pampa Dos Cruces and Pampa Camarones at 1000 and 1010 m, respectively) in the Atacama Desert, extending up to 1500 m in the foothills [22]. Although these species are representatives of the local desert flora, there is no further information on the anatomy of the leaf epidermis, spatial distribution, density of stomata, and trichomes in terrestrial and saxicolous atmospheric plants. This work aims to contribute to the knowledge of the leaf epidermis anatomy of *Tillandsia marconae*, *T. landbeckii*, and *T.virescens*. 

## 2. Results

**Figure 2:**
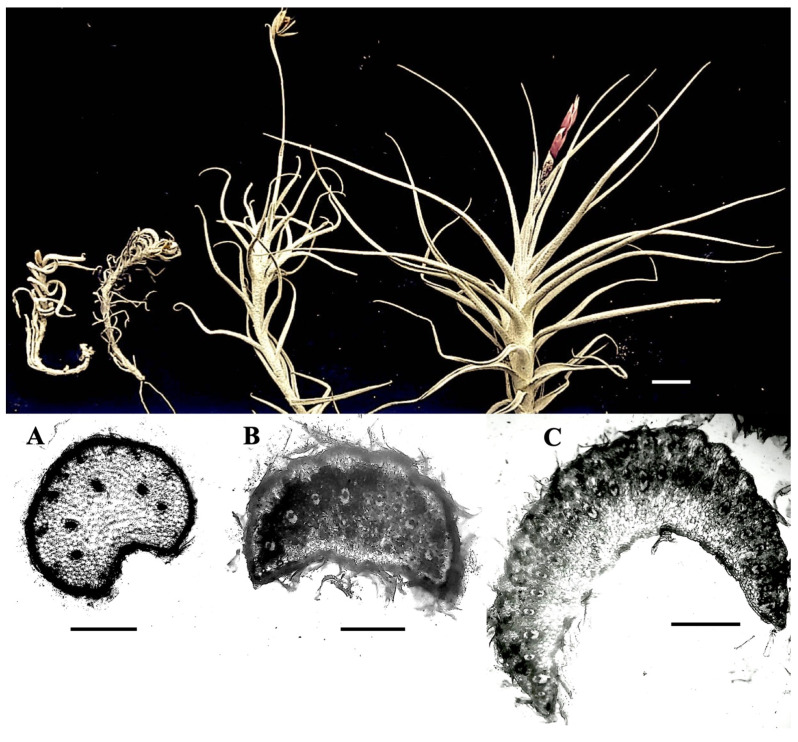
*Tillandsia* leaf, up: natural expansion, down: cross-section. (**A**). *Tillandsia virescens*. (**B**): *Tillandsia landbeckii*, (**C**): *Tillandsia marconae*. Black bars: 1 mm. White bar: 10 mm.

Description. The three species studied have flexuous stems, with rosette foliage and short internodes, whole simple entire leaves, imbricate arranged, clustered, and forming basal rosettes, parallel-veined, sessile, with basal sheaths overlapping. Leaves are long lanceolate and smooth-edged, conduplicate, with a clamping base, wider than the rest of the leaf blade, densely and more or less evenly covered with trichomes on both sides of the leaves (lepidote leaves); stomata only occur on the abaxial surface (hypostomatic leaf) (Figure 1 and Figure 2).

**Figure 3:**
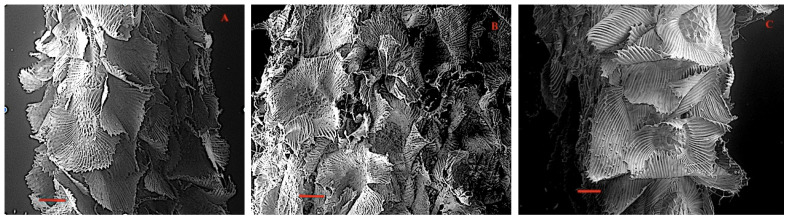
Details of the peltate scale trichomes: (**A**). *Tillandia landbeckii*, (**B**). *T. marconae*, (**C**). *T. virescens*. Bar = 100 µm.

The anatomy of the leaf epidermis presents ordinary epidermal cells, stomata with no subsidiary cells (anomocytic type), and squamiform trichomes [25]. These are formed by a short stalk of a couple of living cells embedded in the leaf tissue with the upper one level with the epidermis, centermost connected to four equal-sized cells, with differentially thickened walls. These are surrounded by concentric rings of dead cells, the innermost of eight, the second one of sixteen cells, all without cytoplasm. A peripheral series of multiple cells similar to each other, without cytoplasm, one cell thick, and long and narrow, form an asymmetric veil with a free apex (Figure 3, Table 1). They are evenly distributed over the leaf blade, with overlapping veils avoiding the exposure of the epidermal tissue. Trichomes are non-glandular, multicellular, and vary in the thickness of their cell walls. The asymmetry is mainly expressed in the margins and base of the leaf blade [25].

*Tillandsia marconae* presented significantly longer leaves than *T. landbeckii* and *T. virescens* and, at the same time, it is the one that presents a veil constituted by a more significant number of cells. Of the three species, *T. virescens* has the shortest leaves, but the trichomes occupy a larger leaf area (Table 1 and Table 2).

The three species showed a higher density of trichomes in the basal portion of the adaxial side (Duncan *p* < 0.05). In *T. marconae*, the lowest density was in the distal portion of the adaxial side of the leaf (Duncan *p* < 0.05) with similar values in the abaxial side, homogeneous values in *T. marconae* and *T. virescens*, but not in *T. landbeckii*. The trichome count considered the central disc and two concentric rings since the flexible wing is removed when applying the “fingernail polish” technique. 

The density of stomata in hypostomatic leaves varied along the leaf blade, and the three species presented significantly higher density in the basal portion of the abaxial side (Duncan *p* < 0.05). In turn, *Tillandsia virescens* and *T. marconae* presented lower density in the middle portion of the abaxial side (Duncan *p* < 0.05).

Although the structural similarity of the three species is evident, the main differences recorded correspond to the thickening of the cell walls of the central disc and the veil (aspects not recorded), which is reflected in the degree of integral flexibility of the wing, being more rigid in *T. virescens* (Figure 2a–c) with respect to *Tillandsia landbeckii* and *T. marconae*. In parallel, the scale size of the peltate trichomes showed significant differences. The scale area in *T. landbeckii* was smaller, while *T. marconae* had an intermediate-sized area, and *T. virescens* showed a more extensive scale area (Table 1). This scale area difference could be related to water availability in the atmosphere and represent an adaptive character of each species.

**Figure 4:**
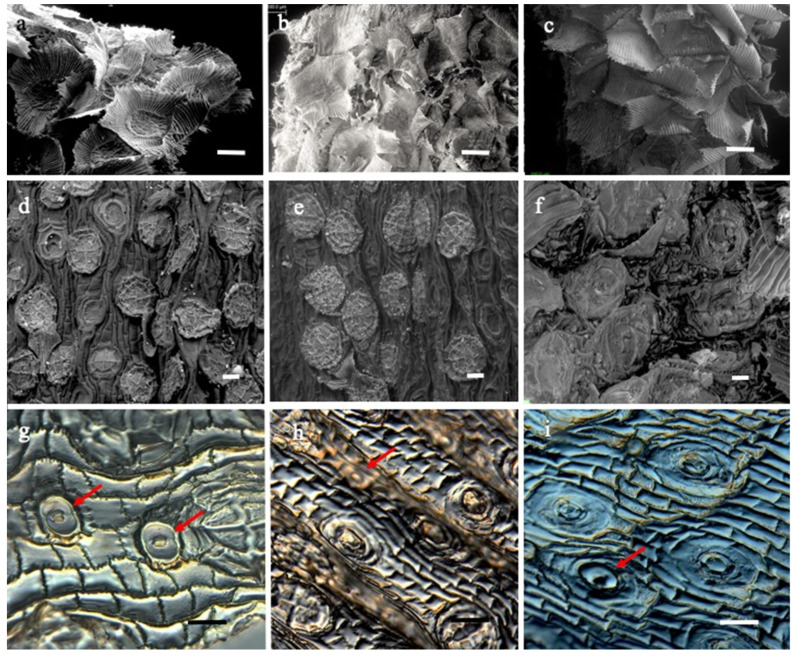
Peltate scales and stomata of *Tillandsia landbeckii* (**a**,**d**,**g**), *T. marconae* (**b**,**e**,**h**), and *T. virescens* (**c**,**f**,**i**). First row: peltate trichomes (**a**–**c**). Second row: trichome base shield: central disk of four cells and two concentric rings of 8 and 16 cells, respectively (**d**–**f**). Third row: “fingernail polish” treatment of the epidermis showing stomata and ordinary epidermal cells (**g**–**i**). Scanning electron microscopy (**a**–**f**); light microscopy (**g**–**i**). Arrows indicate stomata. Bars: (**a**–**c**) = 100 µm; (**d**–**f**) = 40 µm; (**g**–**i**) = 50 µm.

*TILLANDSIA* LANDBECKII. It presents peltate trichomes on both foliar sides; thickened walls of the four central cells and the concentric rings of eight and sixteen cells perfectly delimit the central disc. An asymmetric veil composed of 65 cells forms a flexible wing that overlaps with the wings of neighboring trichomes (Figure 4). The apex of the veil cells is free. Stomata without attached cells is only present in the abaxial epidermis in low density (Table 1, Figure 4a,d,g).

*TILLANDSIA* MARCONAE. The structure of the peltate trichomes is structurally similar to *T. landbeckii* and *T. virescens* and is present on both leaf sides. The four cells of the central disc and all the cells form the first and second concentric rings, which show weakly thickened cell walls (Figure 2c and Figure 4e). The asymmetric flexible shield composed of 85 elongated cells form the wings; several cells are more elongated than others, always leaving the apex of each cell-free (Figure 4b,e,h). Anomocytic stomata occur in low densities (Table 1).

*TILLANDSIA* VIRESCENS. It presents silvery peltate trichomes structurally similar to *T. landbeckii* and *T. marconae*. The upper walls of the four cells of the central disk and those of all the concentric rings are heavily thickened (Figure 4c,f,i), thicker than the walls of the central shield cells of *T. landbeckii* and *T. marconae.* An asymmetric veil with flexible, overlapping wings is formed by multiple elongated cells [27], which are compact and homogeneous with the last third coiled (Figure 4c,f,i). Anomocytic stomata occur in the abaxial side of the blade in low densities (Table 1).

## 3. Discussion 

The genus *Tillandsia* belongs to the subfamily Tillandsioideae (Bromeliaceae), characterized for having originated lepidote leaves with a dense cover of highly specialized peltate trichomes that fulfill functions of foliar absorption of water and nutrients from the environmental humidity. This represents an adaptive, specialized, and indispensable strategy to survive in arid environments and avoid desiccation. They also avoid the negative effect of solar radiation and extreme temperatures [1,9,25,27,28,29]. 

The specimens of the three species studied are atmospheric. *Tillandsia landbeckii* and *T. marconae* are terrestrial and are part of the coastal flora since their survival depends on fog or camanchaca, while *T. virescens* is a saxicolous species from the pre-Puna of the Arica and Parinacota region (Figure 1) that, although it develops short adventitious roots that are often intermingled with lichens (aff. *Parmelia* sp.) and ferns (aff. *Cheilanthes* sp.), these are mainly used to adhere to the rocky substrate on the cliffs where it lives, adopting a hanging life [13,14,22,30]. 

*Tillandsia marconae* and the specimens of *T. landbeckii* used in this study share geographic and environmental components, inhabit a plateau located at 1000 m altitude, grow to form bands or independent cushions (*T. landbeckii* also forms mounds) on a fine sandy substrate in areas with strong wind gusts, many daily hours of sunshine, and dense coastal fog as the only source of humidity. Several authors previously discussed this last component [9,11,14,29,31], who point out that ambient humidity covers the area entirely and is maintained for several hours in the morning and afternoon, preferably during May to August. 

*Tillandsia virescens* is saxicolous in habit and develops profuse fine and short extensions, roots that function only to adhere to the substrate. They are individual plants hanging from almost vertical rocky cliffs, in a prepuneño habitat, at 3000 m, growing on bare rock or next to ferns and lichens. The relative humidity increases from October to March and may receive rainwater precipitation in the summer months [29,32]. 

The influence of the microenvironment on plants can be observed at the morphological and anatomical level, studying the composition of trichomes and the structure and arrangement of stomata. Cach-Pérez et al. [33] demonstrated that epiphytic bromeliads are sensitive to environmental variations, such as water availability, light, or environmental temperature, generating morpho-physiological changes [28,34]. Similar data are lacking for terrestrial atmospheric and saxicolous species. 

The three species studied grow under xeric conditions and show similarities in the morphology of the vegetative characters studied and are related to the survival strategy in arid environments, such as hypostomatic conduplicate leaves, the low density of anomocytic stomata (without attached cells), and peltate trichomes. Trichome shields are flat, one cell thick, with an orderly arrangement of empty cells. Centermost, just over the point where the distal stalk cell is connected to the underside of the *Tillandsia* shield, are four equal-sized, thick-walled cells, which comprise the central disk, surrounded by two concentric rings of eight and sixteen, and a peripheral series of long and narrow cells forming a flexible and asymmetric wing or veil, with the apical end free (Figure 1a–c and Figure 3). The second ring functions as a hinge, allowing the wing to bend upward when dry [3,25]. 

The wing cells are homogeneous, in an unstratified arrangement, present an irregular outline, and form a dense protective layer for the isolated stomata by completely covering the epidermal tissue due to the overlapping of the neighboring trichome veils. The size and number of cells of the trichome veil are different for each species [9,25], (Table 1). Benzing et al. [3] reported that in the case of the atmospherics *Tillandsia circinata* and *T. tectorum*, even when the shields are flexed, the stomates are covered at all times [3], allowing us to understand why the density of stomata in the three species studied (*T. landbeckii*, *T. marconae*, and *T. virescens*) is so low (Table 2). 

With the vegetal material that was studied, it was expected to find similarities between *Tillandsia landbeckii* and *T. marconae* regarding the density of trichomes and stomata, since they share environmental components, such as inhabiting a wide plateau located at a thousand meters of altitude, with a fine sandy substrate and regular coastal fog as a source of humidity [31]. The higher density of trichomes of *T. landbeckii* can be explained because the plateau it inhabits is extensive, and its only source of humidity is sporadic fog, limited to a couple of months, strong gusts of wind, and many hours of sunshine. It was expected to find differences between these two species and *T. virescens* in that the latter is saxicolous, and thus it develops an extensive root branching of fine and short extensions to support the substrate. It hangs on almost vertical rocky cliffs several meters high, inhabiting another altitudinal level in the pre-Puna, at three thousand meters of altitude, intermingled with ferns (aff. *Cheilanthes* sp.) and lichens (aff. *Parmelia* sp.) (Belmonte, unpublished data), and its source of moisture is not the camanchaca, as this does not exceed 1500 m altitude.

**Figure 5:**
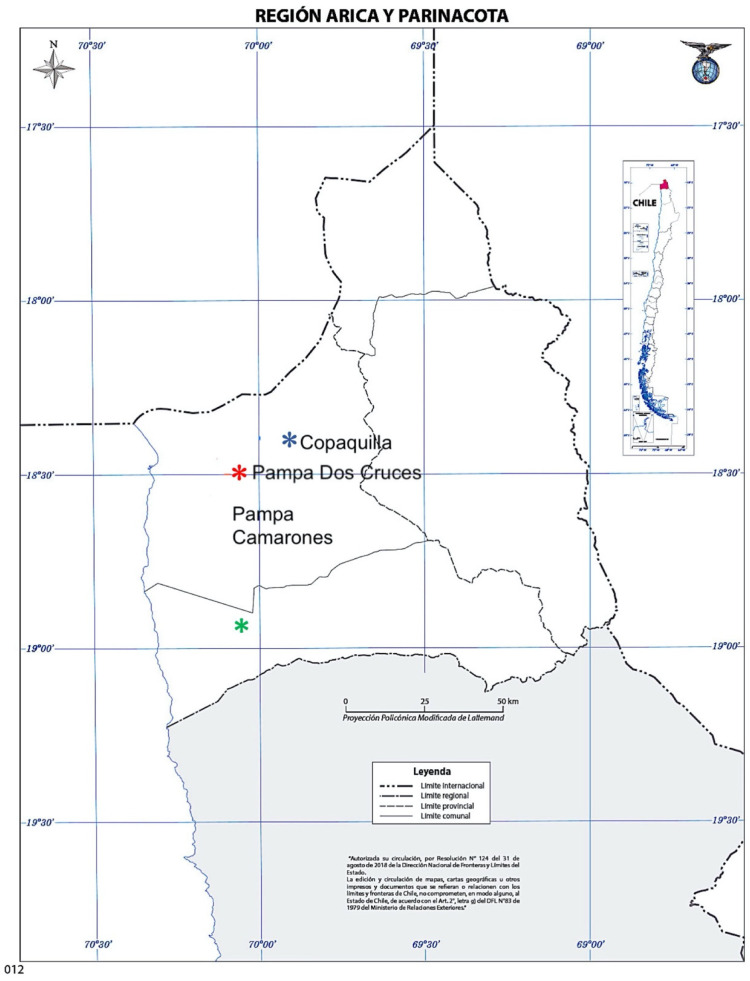
Sampling sites of the 3 species of *Tillandsia* in the region of Arica and Parinacota. 1. 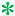 *Tillandsia landbeckii*, Pampa Camarones, 1010 m; 2. 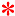 *T. marconae*, Pampa Dos Cruces, 1000 m; and 3. 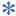
*T. virescens*, Pukara de Copaquilla, 3050 m. (Source: Google Earth).

Images of the peltate trichomes obtained with SEM reflect features specific to each species, given by the thickness of the external walls of the cells that form the central shield and the concentric rings that surround it. These cells have no cytoplasm, and their walls are differentially impregnated with cutin and pectin. The wings are formed by empty cells whose outer cellulose walls are impregnated with pectin and cutin [3,9,25], which is reflected in the degree of inflection of the veil, which adopts an undulatory movement in *Tillandsia landbeckii* and *T. marconae* (Figure 4a,b and Figure 5). Nevertheless, this could only affect the outer third of the veil cells (data not shown), a situation that may account for the composition of the cell wall. The trichome of *T. virescens’* (Figure 3) silvery appearance has been described for other *Tillandsia* species [9] and is associated with the cutinization of its walls. Benzing and collaborators [3] argue that in trichomes with a flexible veil, the wings extend over the leaf surface when hydrated, but when dry, they flutter upwards, in a phenomenon of epinasty, thus increasing their reflective capacity. This movement is contributed to by the two concentric rings of eight and sixteen cells surrounding the central shield [30].

The data submitted are consistent with previous information [9,28,34], allowing us to present the idea that the structure of the peltate scale on the epidermal surface is a conserved trait in the species studied, establishing that the altitudinal or elevational gradients modify the external structures, such as leaf size, trichome and stomata density, as well as leaf thickness, mitigating the stressful conditions typical of these zones [35,36,37].

## 4. Materials and Methods

SELECTION OF *TILLANDSIA* POPULATIONS

*Tillandsia landbeckii* of Pampa Camarones. Pampa Camarones is an intermediate plain between the Chaca ravine and the Camarones ravine, 100 km south of the city of Arica, on the northern border of Chile (Table 3, Figure 5). Geomorphologically, it corresponds to a plateau of the Llano of fluvial and alluvial sedimentation, Pampitas, and the Cordillera de la Costa, which has eolian deposits as a geomorphological unit, where there are areas of deposits in two formats: in large sections as dune accumulations stabilized by the presence of *T. landbeckii*, and by depressions with the accumulation of sand by gravity and stagnation [38]. In Pampa Camarones, *T. landbeckii* forms a monospecific community that grows in small, isolated cushions that develop on sandy substratum or wavy structure mounds that can reach over two meters high and several meters long (Figure 1a). These mounds are formed by the stratigraphic combination of horizontal bands of plant material alternating with the sterile substrate; the mounds are distanced without forming grouped units. *T. landbeckii* does not form these mounds in Pampa Dos Cruces but grows around cushions or bands of *T. marconae* (Figure 1b) or forms isolated cushions or bands.

*Tillandsia marconae* from Pampa Dos Cruces. From a conservationist point of view, this is a site declared a priority by the Ministry of National Assets (Decree 573 2010) because it is the only locality described in Chile for *T. marconae* (Table 2). In this area, locally called “Calanchucal”, the dominant species is *T. marconae*, which grows in short horizontal crawling bands measuring between 150 and 750 cm long, 40 and 90 cm wide, and 17 and 38 cm high. In the area closest to the Lluta Valley, 30 km northeast of the city of Arica (Figure 5), it shares a habitat with *T. landbeckii*, which forms small, isolated cushions or grows around bands of *T. marconae* [13,14]. Pampa Dos Cruces is a plateau located at 1000 m near the town of Poconchile, in the highest part, close to the Lluta valley. 

*Tillandsia virescens* from the Copaquilla cliffs. It occurs as individual saxicolous plants that form small hanging cushions on rocky cliffs surrounding the Copaquilla River, Putre Province, Arica and the Parinacota Region, a watercourse that spends at least 10 months dry. The cushions are attached to the substrate by short, adventitious roots, which intertwine with ferns (aff. *Cheilanthes* sp.) and lichens (aff. *Parmelia* sp.) (Figure 1c and Figure 5).

Material collected. The samples corresponded to whole plants collected in their natural environment (Pampa Camarones, Pampa Dos Cruces, and Copaquilla cliffs), stored in paper bags, and taken to the Plant Pathology Laboratory of the Universidad de Tarapacá, Arica, Chile, for study. Ten specimens of each species were randomly selected, considering similar individuals within the population in terms of vigor, vegetative state, and environmental distribution.

Optical microscopy. The indexed leaf samples were studied with an optical microscope (Olympus mod. SZX-7). Photographs were recorded (Microimaging CCD-5.1), attached to the microscope, and registered in the Micrometrics program (SE Premium). The fingernail polish technique [39] consisted of removing the wings of the trichomes with transparent adhesive tape to clean the surface and expose the epidermal cells to study details of the leaf epidermal cells. Then, four successive layers of clear nail polish were applied, and once dry, these layers were peeled off, achieving an impression of the bare leaf surface. The dried enamel layer was mounted on a slide and observed under an optical microscope. For each species studied, 10 visual fields of the adaxial and abaxial leaf epidermis, selected from the apical, middle, and basal zones of the leaves, were randomly recorded. The morphology of the central shield cells, the two surrounding rings, the veil cells of the peltate trichomes, stoma occluding cells, and epidermal cells of the three *Tillandsia* species collected were studied. The density of stomata and trichomes (n/mm^2^) was calculated in a 400× field area [40,41].

Scanning electron microscopy (SEM). The observations with SEM (Zeiss mod. EVO LS-10) were made with direct mounting, without coating. The specimens were prepared in aluminum sample holders with double contact carbon adhesives and were analyzed in variable pressure (VP) mode, where the chamber pressure was 150 Pa (low vacuum) and in the column 2 × 10^−5^ Torr (high vacuum). The working distance (WD) that was used varied depending on the type of sample and what needed to be observed; we worked with an accelerating voltage of 15 KV and inclination from 0° to 90°, while the images were taken with 3024 × 2304 pixel resolution at a scanning speed of 12 min and 54 s. In basal and medial positions, two samples per species were analyzed, both from the beam and the underside. The work was carried out at the Bioarchaeology Laboratory of the High Research Institute of the University of Tarapacá, Arica, Chile.

Statistical analysis. All results were subjected to normality tests, analysis of variance, and Duncan’s multiple comparisons of the means test to determine significant statistical differences between species. We worked with R software [26].

## Figures and Tables

**Table 1:** Leaf anatomical comparison between *Tillandsia landbeckii*, *T. marconae,* and *T. virescens*. Duncan’s test (*p* < 0.05). Equal letters represent statistical equality [26]. OCd = ordinary cell density; Sd = stomatal density; Td = trichome density; PTa = peltate trichome area (µm^2^); Lw = leaf width (mm); Ll = leaf length (mm); La = leaf area (mm^2^); NVc = number of veil cells.

	OCd	Sd	Td	PTa (µm^2^)	Lw (mm)	Ll (mm)	La (mm^2^)	NVc
Abaxial side								
*T. landbeckii*	1.9 × 10^−2^ b	3 × 10^−4^ a	1.22 × 10^−3^ b	66048.5 a	2.3 a	42.9 a	96.8 a	65 a
*T. marconae*	1.9 × 10^−2^ b	1 × 10^−4^ a	0.87 × 10^−3^ a	105114.2 b	9.4 b	110.8 b	1040.6 b	85 b
*T. virescens*	1.6 × 10^−2^ a	1 × 10^−4^ a	1.01 × 10^−3^ a	174475.8 c	2.1 a	20.4 c	42.1 c	62 a
Adaxial side								
*T. landbeckii*	1.6 × 10^−2^ a	0 a	1.13 × 10^−3^ a	---				
*T. marconae*	1.7 × 10^−2^ a	0 a	0.97 × 10^−3^ a	---				
*T. virescens*	1.7 × 10^−2^ a	0 a	1.08 × 10^−3^ a	---				

**Table 2:** Anatomic comparison between *Tillandsia marconae*, *T. landbeckii,* and *T. virescens*.

	*T. marconae*	*T. landbeckii*	*T. virescens*
Pyllotaxis	Rosette	Rosette	Distichous
Conduplicate leaf	The whole blade	The whole blade	The first basal third is conduplicate and becomes cylindrical towards the apex
Marginal configuration	Crenate	Crenate	Entire
Epidermis	Uniseriate with thick homogenous walls rectangular in shape and straight anticlinal walls	Uniseriate with thick homogenous walls rectangular in shape and straight anticlinal walls	Uniseriate with thick homogenous walls rectangular in shape and straight anticlinal walls
Ground parenchyma: Hypodermis	Hypodermal layers of large, thin-walled, isodiametric parenchyma cells that store water are present close to the abaxial leaf surface. On the opposite side, the hypodermis forms isolated clusters of a few enlarged cells	Close to the abaxial leaf surface, hypodermis is present in the form of 2–3 layers of thin-walled, isodiametric parenchyma cells. On the opposite side, the hypodermis forms wide isolated clusters of enlarged cells	Globose translucent isodiametric parenchyma cells and chlorophyll-bearing cells are homogeneously distributed throughout the area
Ground parenchyma: Chlorenchyma	The chlorenchyma is formed by angular, isodiametric chlorophyll-bearing cells, isolated from the abaxial leaf surface by many layers of water-storage cells	The chlorenchyma is formed by angular, isodiametric chlorophyll-bearing cells, isolated from the abaxial leaf surface by a couple of hypodermal layers of water-storage cells	Chlorophyll-bearing and water-storage cells are intermingled
Vascular bundles	The vascular bundles are distributed in a linear series following the shape of the leaf, in the area occupied by chlorophyll-bearing cells	The vascular bundles are distributed in a linear series following the shape of the leaf, in the area occupied by chlorophyll-bearing cells	The vascular bundles are distributed throughout the area in the middle of the ground parenchyma, in a double concentric series.
Bundle sheath	Bundle sheath one cell thick, surrounded by a great amount of thick-walled schlerenchyma cells	Bundle sheath one cell thick, surrounded by a great amount of thick-walled schlerenchyma cells	Double bundle sheath; the inner one formed by small cells and the outer one formed by large cells without chlorophyll of the ground parenchyma
Stomata	Stomata are anomocytic, present only in the abaxial epidermis.	Stomata are anomocytic, present only in the abaxial epidermis.	Stomata are anomocytic, present only in the abaxial epidermis.
Scales	Peltate trichome in both leaf surfaces 4 + 8 + 16 + 85 (disc cells, ring cells, veil)	Peltate trichome in both leaf surfaces 4 + 8 + 16 + 65 (disc cells, ring cells, veil)	Peltate trichome in both leaf surfaces 4 + 8 + 16 + 62 (disc cells, ring cells, veil)

**Table 3:** Location of *Tillandsia landbeckii*, *T. marconae*, and *T. virescens* (Atacama Desert, Chile).

Species	Location Altitude (m)	Geographical Coordinates		Collection Date
		S	W	
*T. landbeckii*	Pampa Camarones1010	18°52′29.66″ S	70°7′10.85″ W	21 July 2017
*T. marconae*	Pampa dos Cruces1000	18°28′43.48″ S	70°5′16.69″ W	21 July 2017
*T. virescens*	Pukara de Copaquilla3050	18°23′34.49″ S	69°38′32.19″ W	14 March 2018

## Data Availability

No new data were created or analyzed in this study. Data sharing is not applicable to this article.
